# Supplementary material for: Identification of a Selective RelA Inhibitor Based on DSE-FRET Screening Methods
Source: Int J Mol Sci. 2020 Nov 30;21(23):9150. doi: 10.3390/ijms21239150 (PMC7734590; doi:10.3390/ijms21239150)
Supplement: Supplementary file 1 [file ijms-21-09150-s001.zip › Supplementary_Figure_S1__S2__S3__S4_112720_Fri_2.pdf]

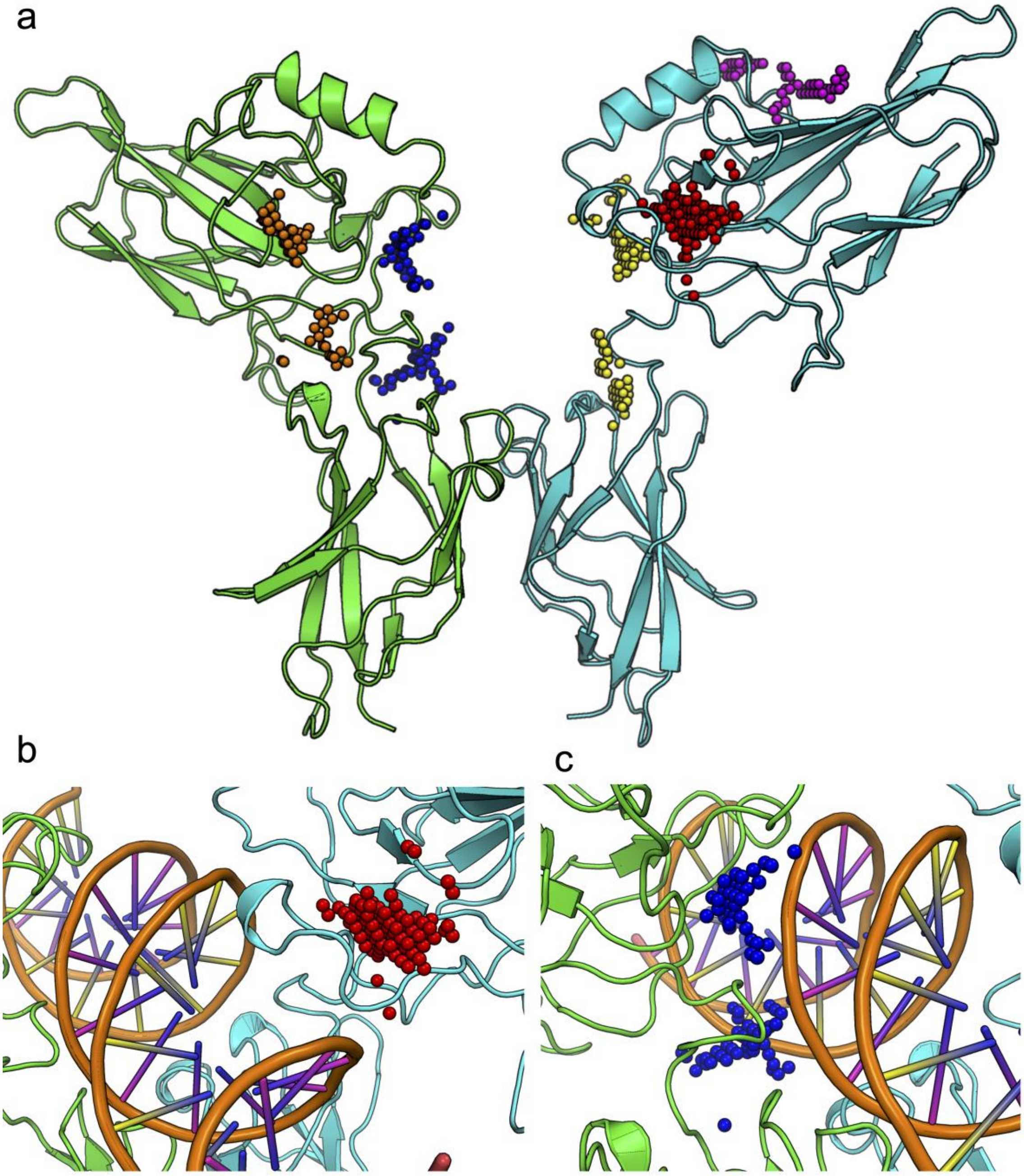

Supplemental Figure S1. Identification of putative small molecule binding pockets on the surface of NF-κB p65 (RelA) homodimer. Colored spheres shown in (A) represent site points belonging to a particular pocket. Red, yellow, blue, magenta, and orange represent the top 5 binding pockets, respectively, with Sitescore values in decreasing order. (B) and (C) show the close-up of binding pocket 1 (red) and 3 (blue), respectively. DNA molecule is also shown in the figure.

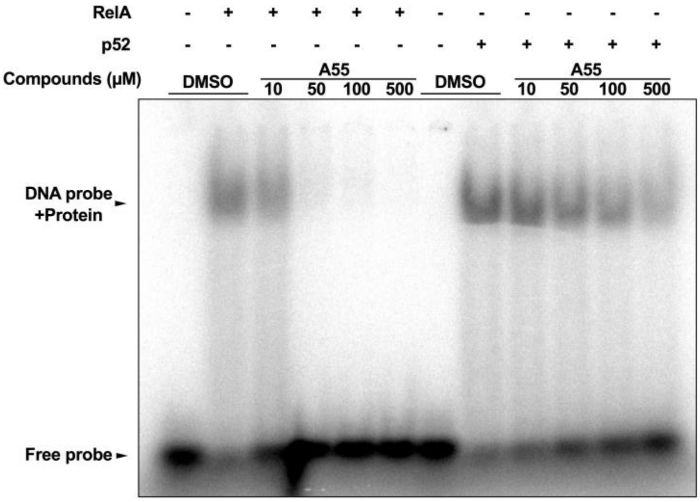

Supplemental Figure S2. EMSA with recombinant RelA or p52 were conducted using radiolabeled probes containing the NF-κB binding site.

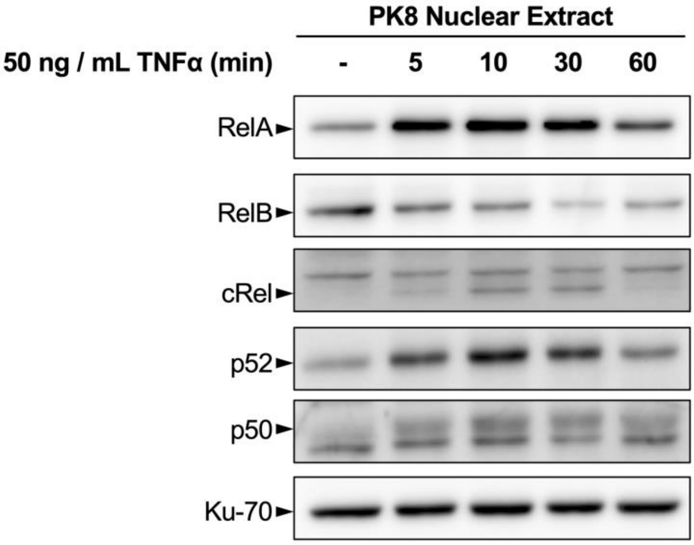

Supplemental Figure S3. NF-kB proteins of PK8 nuclear extract. PK8 cells were treated with TNFα (50 ng/mL) for 5 – 60 min; nuclear protein was then extracted. Ku-70 was used as loading control. EMSA was performed using PK8 nuclear extract of TNFα 10 min treatment.

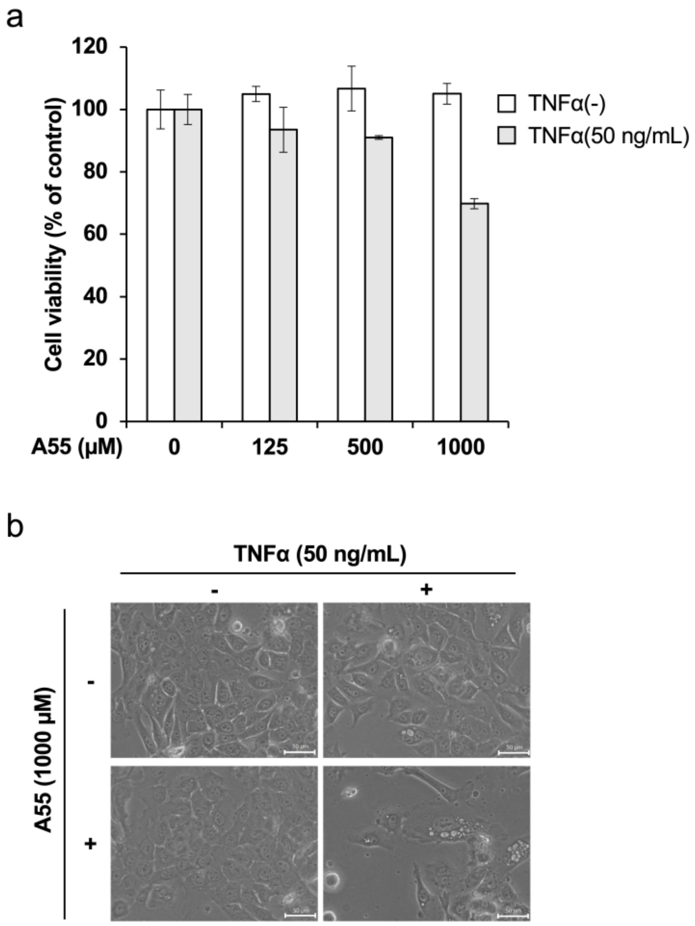

Supplemental Figure S4. Cell viability assay for PK8 cells treated with TNFα (50 ng/ mL) for 48 hr in the presence or absence of A55. (a): Cell viability was measured using Presto blue assay. TNFα(-) in the absence of A55 was used as a control. Error bars represent SD (n = 3) (b): Cell morphology.
